# Mangroves provide blue carbon ecological value at a low freshwater cost

**DOI:** 10.1038/s41598-022-21514-8

**Published:** 2022-10-21

**Authors:** Ken W. Krauss, Catherine E. Lovelock, Luzhen Chen, Uta Berger, Marilyn C. Ball, Ruth Reef, Ronny Peters, Hannah Bowen, Alejandra G. Vovides, Eric J. Ward, Marie-Christin Wimmler, Joel Carr, Pete Bunting, Jamie A. Duberstein

**Affiliations:** 1 U.S. Geological Survey, Wetland and Aquatic Research Center, Lafayette, LA 70506 USA; 2 School of Biological Sciences, The University of Queensland, Brisbane, 4072 Australia; 3 Key Laboratory of the Ministry of Education for Coastal and Wetland Ecosystems, College of the Environment and Ecology, Xiamen University, Xiamen, 361102 Fujian China; 4 Institute of Forest Growth and Forest Computer Sciences, Technische Universität Dresden, 01062 Dresden, Germany; 5 Research School of Biology, The Australian National University, Acton, ACT 2601 Australia; 6 School of Earth, Atmosphere and Environment, Monash University, Clayton, VIC 3800 Australia; 7 Instituto de Ecología AC, Carretera antigua a Coatepec 351, 91073 Xalapa, Veracruz Mexico; 8 School of Geographical and Earth Sciences, University of Glasgow, Glasgow, UK; 9 U.S. Geological Survey, Eastern Ecological Science Center, Laurel, MD 20708 USA; 10 Department of Geography and Earth Sciences, Aberystwyth University, Aberystwyth, Wales UK; 11 Baruch Institute of Coastal Ecology and Forest Science, Clemson University, Georgetown, SC 29442 USA

**Keywords:** Ecophysiology, Forest ecology, Wetlands ecology

## Abstract

“Blue carbon” wetland vegetation has a limited freshwater requirement. One type, mangroves, utilizes less freshwater during transpiration than adjacent terrestrial ecoregions, equating to only 43% (average) to 57% (potential) of evapotranspiration ($ET$). Here, we demonstrate that comparative consumptive water use by mangrove vegetation is as much as 2905 kL H_2_O ha^−1^ year^−1^ less than adjacent ecoregions with ${E}_{c}$-to-$ET$ ratios of 47–70%. Lower porewater salinity would, however, increase mangrove ${E}_{c}$-to-$ET$ ratios by affecting leaf-, tree-, and stand-level eco-physiological controls on transpiration. Restricted water use is also additive to other ecosystem services provided by mangroves, such as high carbon sequestration, coastal protection and support of biodiversity within estuarine and marine environments. Low freshwater demand enables mangroves to sustain ecological values of connected estuarine ecosystems with future reductions in freshwater while not competing with the freshwater needs of humans. Conservative water use may also be a characteristic of other emergent blue carbon wetlands.

## Introduction

Water will be among the most limiting natural resource of the future, influencing ecological flows among coastal environments and soon affecting at least 80% of the world’s population directly^1,2^. Competition for water between humans and natural ecosystems is critical in driving outcomes for natural ecosystem health, and water supply is projected to increasingly shift toward human use^3^. Selection of “nature-based solutions” to sequester carbon or enhance climate resilience could simultaneously consider the water economy of the ecosystem doing the work. To that end, “blue carbon” wetlands may have emergent but yet unrecognized ecological value.

Blue carbon wetlands comprise a variety of coastal ecosystems – most notably mangroves, saltmarshes and seagrasses^4 ^– but also include upper estuarine tidal wetlands, and adjacent ecosystems such as salt flats and macroalgal communities^5^. As a group, they provide tremendous value to human societies^6,7^. Among these, blue carbon wetlands store large amounts of carbon in aboveground biomass and in soils as their roots trap sediments and expand soil volumes^8,9^. As a result, blue carbon wetlands have increasingly become important for environmental management of coasts and for use as self-sustaining nature-based solutions in adapting coastlines to rising seas^10^.

Mangrove vegetation is located within the intertidal zone^11^ and tolerates but does not always require saline water^12^ (but see ref.^13^). Mangroves extract freshwater from ultrafiltration of seawater through root tissue^14,15^ and consume freshwater differentially when it is readily available (groundwater, rainfall at low tide)^16,17^. Once water is absorbed by roots and the metabolic costs of excluding salt are incurred, bulk water transport from roots-to-leaves is driven by pressure gradients created as water is transpired^18^. Especially under saline soil conditions, mangroves have been recognized as having high plasticity in adjusting their rates of leaf-level water use efficiency to accommodate osmotic gradients^19^.

Of 214 evaluations (number of species × number of studies) of leaf-level water-use efficiency published over the last three decades (source: Scopus/Web of Science), most confirm what was originally presented from a 1989 field study^20^, that intrinsic leaf-level photosynthetic water use efficiency (${WUE}_{int}$: ratio of leaf-level net photosynthetic rate to stomatal conductance to water vapor) becomes higher as the salinity of water within mangrove roots increases (Fig. S1). This relationship is seemingly amplified by high atmospheric vapor pressure deficit; however, leaf-level marginal water costs [i.e., the ratio of change in actual water used (water cost) vs. change in photosynthetic rate (carbon gained)] are not strongly influenced by atmospheric moisture or temperature changes over diel cycles for mangroves^21^. Nevertheless, high rates of water use efficiency at the leaf-level have promoted the idea that mangroves are able to use less water as stress gradients increase. Indeed, salinity is one of those stressors that affect both ${WUE}_{int}$ and marginal water costs in ways that may also influence water use by whole trees and canopies.

In this study, we assess whether one type of blue carbon wetland – mangroves – may be particularly efficient in the amount of water they use at leaf, tree, and ecosystem scales during the process of atmospheric carbon capture^15^. Mangroves can, in many locations, grow with limited freshwater availability^17^, their roots extracting freshwater from seawater^14^, and they can obtain some freshwater directly through foliage^22–25^. We explore three questions. First, are leaf-level and tree-level water use strategies in mangroves similarly conservative? Second, how much water does mangrove vegetation use? Third, is mangrove vegetation water use low compared to water use from non-wetland ecosystems (e.g., terrestrial forests) or from vegetation that arises from the conversion of mangroves to alternative land uses (e.g., oil palm plantations)? We answer these questions by exploring a pulse of recent studies and model development that allow insight across scales.

## Results

**Figure 1:**
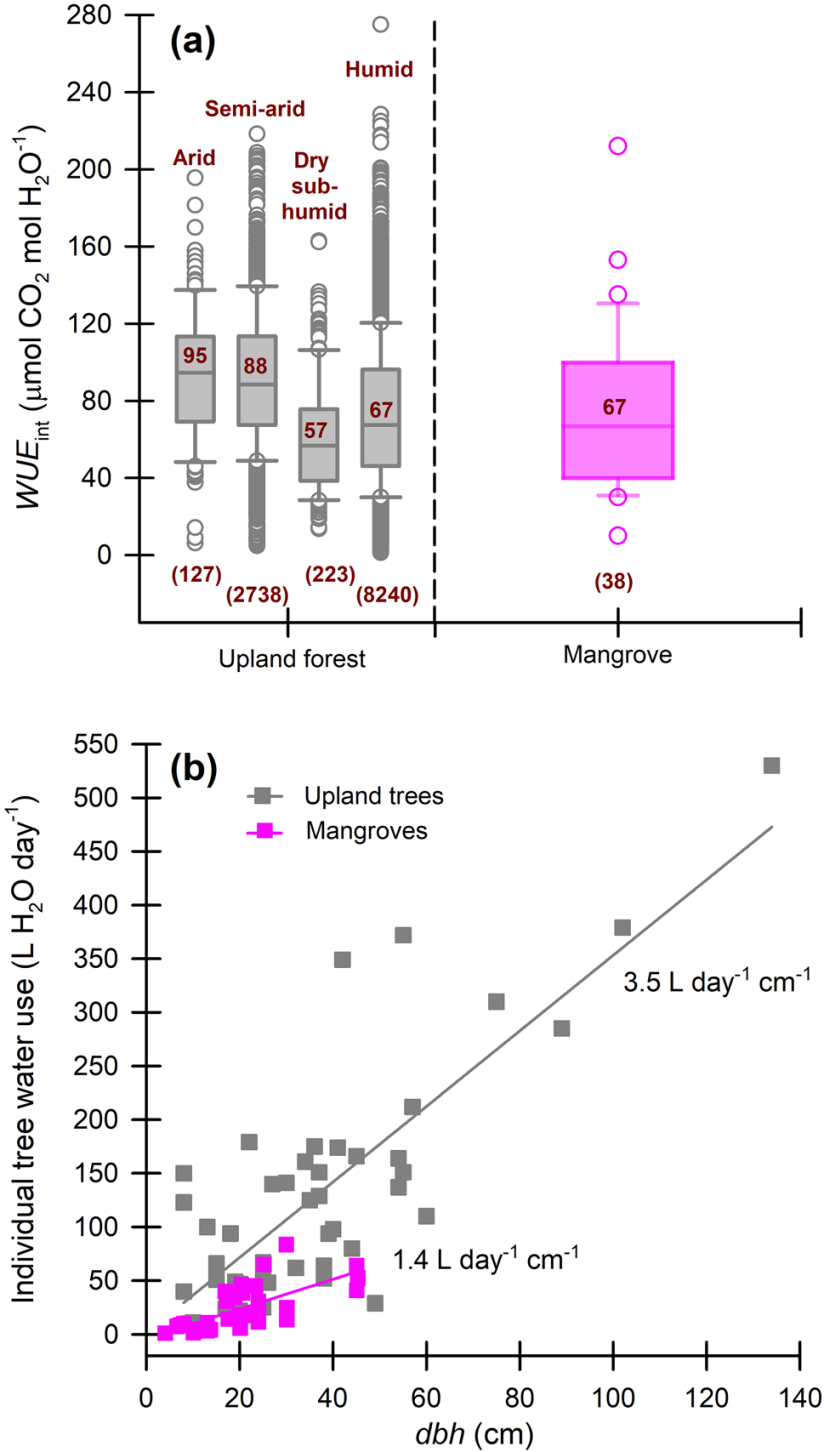
Leaf-intrinsic water use efficiency ( ${WUE}_{int}$ ) and individual tree water use for mangroves versus terrestrial woody vegetation. (**a**) ${WUE}_{int}$ for a mix of seedlings, saplings, and trees of upland species from arid, semi-arid, dry semi-arid, and humid environments^60^ versus mangrove, depicting median (center line and values), upper and lower quartiles (box limits), 1.5 × interquartile ranges (whiskers), and outliers (points) as box-and-whisker plots, and sample sizes depicted within parentheses. (**b**) Individual tree water use (median) from sap flow studies conducted on mangrove trees relative to diameter at breast height (*dbh*), with comparison to upland trees (from ref.^61^).

Mangrove water use efficiency is reported over a range of scales; as long-term, instantaneous, or intrinsic, and at the leaf, plant, or ecosystem level, each necessitating a range of assumptions. This 3 × 3 matrix of scale is dominated in the mangrove literature by leaf-level ${WUE}_{int}$, with fewer assessments of water use efficiency at other spatial or temporal scales. In our analysis, median ${WUE}_{int}$ of terrestrial woody plant species ranged from 57 μmol CO_2_ (mol H_2_O)^−1^ from dry sub-humid environments to 88–95 μmol CO_2_/(mol H_2_O) in arid and semi-arid environments (Fig. 1a). Median ${WUE}_{int}$ for mangrove leaves was 67 μmol CO_2_/(mol H_2_O)^−1^ (range, 10 – 212) which is only slightly higher than shrubs and trees from dry sub-humid environments (*p* < 0.05, Dunn’s ranked sums).

However, lack of differentiation among comparative water use efficiencies at the leaf-level did not scale. Instead, upland forest trees used, on average, 3.5 L H_2_O day^−1^ cm^−1^ of *dbh* (diameter at breast height, ~ 1.3 m above ground) while mangrove trees used only 1.4 L H_2_O day^−1^ cm^−1^ of *dbh* (Fig. 1b) (statistical *Q* = 6.06, *p* < 0.001, Dunn’s ranked sums). Therefore, from sap flow-derived individual tree water flux data, we can begin to understand organizational scales at which transpirational water loss is most limited from mangrove vegetation.

Scaling further to the canopy, we discovered that slopes of relationships relating canopy transpiration to net primary productivity (${E}_{c}$-to-NPP) for mangroves were similar between locations in China and the US (Fig. S2), despite being developed on sites separated by half the globe. Furthermore, slopes of both relationships differed significantly from zero (*p* < 0.001; r^2^ ≥ 0.955) suggesting the potential to use NPP of focal mangrove forests to estimate ${E}_{c}$ where data on ${E}_{c}$ were unavailable. We developed the following equation:

1 $${E}_{c} = 384.59\left(NPP\right)+33.56$$

where ${E}_{c}$ is in units of mm H_2_O year^−1^ and NPP is in units of kg C m^-2^ year^−1^. Note that 1 mm H_2_O is equivalent to 1 L H_2_O per square meter of ground area.

**Figure 2:**
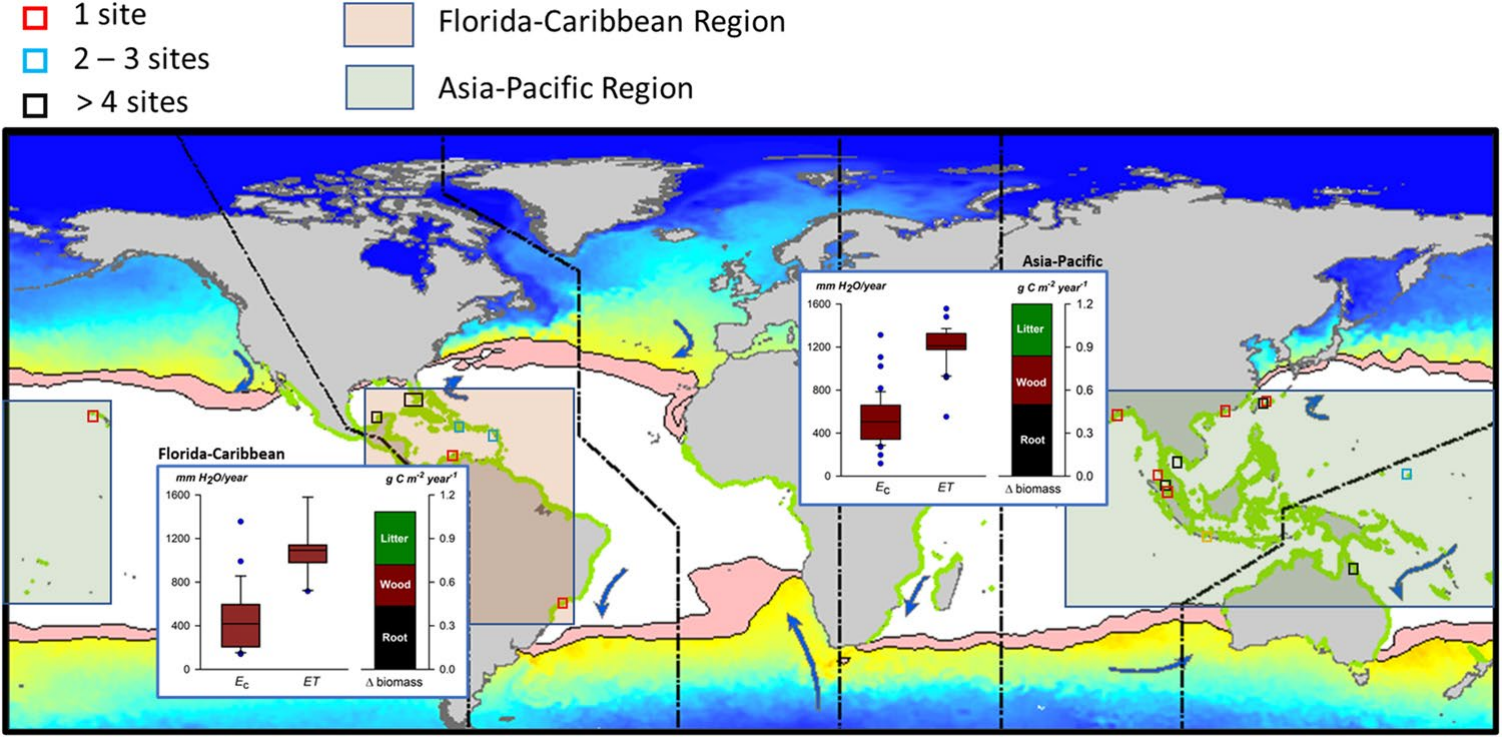
Location of 71 mangrove study sites from the Florida-Caribbean and Indo-Pacific regions from which net primary productivity (NPP, kg C m^-2^ year^−1^) data were used to determine canopy transpiration (${E}_{c}$). Polygons (pink) over the oceans represent 20 °C summer and winter isotherms influencing mangrove distributions (updated from Duke et al. 1998, *Glob. Ecol. Biogeogr. Letts.*, v. 7, p. 27–47). Insets represent comparative average ${E}_{c}$, $ET$, and NPP values for litter, wood, and roots assuming a continuous mangrove coverage versus $ET$ at a scale of 1 km^2^ for each of the two regions. Box plot depictions are the same as in Fig. 1. Base image created using ArcMAP 10 (Esri, Inc., Redlands, California, USA), https://desktop.arcgis.com/en/arcmap/ .

Using data from 26 published records reporting on 71 mangrove study sites located in the Florida-Caribbean Region (N = 24) and the Asia–Pacific Region (N = 47) that had sufficient data to derive approximate NPP values, we used Eq. (1) to estimate what ${E}_{c}$ might be in order to support estimated NPP for those 71 locations (Fig. 2). Average ${E}_{c}$-to-$ET$ ratios for mangroves was 43.4% (± 3.0%, S.E.) when we assume ${E}_{c}$ from sites under study-reported environmental conditions, but as high as 57.4% (± 4.0%, S.E.) when we consider the potential for higher ${E}_{c}$ at sites with lower porewater salinity.

Mangrove ${E}_{c}$-to-$ET$ ratios were approximately 27% less than that of tropical rainforests and about 4% less than that of Mediterranean shrublands (Table 1). Thus, average water use differences between mangroves and other ecoregions ranged from 121 to 2905 kL ha^−1^ year^−1^, or from − 302 to 1399 kL ha^−1^ year^−1^ versus potential water use from mangroves assuming low salinity. Under low salinity scenarios, higher water use by mangroves would occur when compared with temperate coniferous forest, desert, and Mediterranean shrubland. However, the majority of differences in consumptive water use reductions by mangroves are associated with the tropics where 93% of all mangroves occur (Table 1).

**Table 1:** Global summary of water use characteristics for mangrove forests versus the seven relevant ecoregional types reported by Schlesinger & Jasechko^48^. Canopy transpiration of dominant vegetation is abbreviated, *E*_c_, and regional evapotranspiration (from MODIS satellite) is abbreviated, *ET*. "Average" columns assume *E*_c_-to-*ET* ratios as they might occur under current stand conditions reported by the primary reference. "Potential" columns assume *E*_c_-to-*ET* ratios that might occur if mangrove porewaters freshen significantly versus average condition.

Ecoregion	*E*_*c*_/*ET* (%) (± 1 S.D.)		N	*E*_*c*_/*ET* minus Mangrove (%)		*ET* (mm H_2_O year^−1^)	Reduction by mangrove water use by ecoregion (mm H_2_O year^−1^)		Reduction by mangrove water use by ecoregion (kL H_2_O ha^−1^ year^−1^)		Mangrove area associated with ecoregion (ha)	Global reduction in water use by comparative ecoregion (GL H_2_O year^−1^)	
	Average	Potential		Average	Potential		Average	Potential	Average	Potential		Average	Potential
Tropical rainforest	70 ± 14	–	8	27	13	1076	290.52	139.88	2905	1399	11,233,190	32,632	15,713
Temperate deciduous forest	67 ± 14	–	9	24	10	549	131.76	54.90	1318	549	84,468	111	46.4
Tropical grassland	62 ± 19	–	5	19	5	583	110.77	29.15	1108	292	1,474,088	1,633	429.7
Temperate grassland	57 ± 19	–	8	14	0	332	46.48	0.00	465	0	518,259	241	0.0
Temperate coniferous forest	55 ± 15	–	13	12	− 2	458	54.96	− 9.16	550	− 92	32,410	18	− 3.0
Desert	54 ± 18	–	14	11	− 3	209	22.99	− 6.27	230	− 63	324,492	75	− 20.3
Mediterranean shrubland	47 ± 10	–	4	4	− 10	302	12.08	− 30.20	121	− 302	17,020	2	− 5.1
Mangrove	43 ± 26	57 ± 34	71	0	0	1172	0	0	0	0	–	–	–
**TOTAL**												**34,711**	**16,161**

## Discussion

Surface or groundwater seeping through mangroves and out to the ocean is not necessarily available for direct human consumption, but unconsumed freshwater that flows to marine and estuarine environments has inherently high value because of its importance in supporting wetland and coastal ocean productivity, and thus, sustained ecological processes within adjacent estuaries^26^. Given that over 80% of freshwater currently consumed globally by humans is associated with food production^27^, food and water security are intricately linked. For this reason, consumption of marine fish in lieu of crops, for example, has been shown to save as much as 50% of a region’s freshwater resource^28^. Mangroves contribute to this efficiency by supporting marine and estuarine food webs at low freshwater costs. Additionally, unlike many terrestrial ecosystems, mangrove survival can be potentially sustained for short periods of time (albeit at lower productivity) during periodic (or even sustained) reductions in freshwater to the coast^29^, at least to a point^30^.

Mangroves also have lower water use than alternative land uses to which they have been converted. For example, oil palm (*Elaeis guineensis* Jacq.) plantations, have replaced 18,467 ha of mangroves in Indonesia, Myanmar, Malaysia, and Thailand between the years 2000–2012^31^. These plantations use more water through ${E}_{c}$ than the mangroves they have replaced. ${E}_{c}$ from mature oil palm plantations (> 12 years old) range from 53^32^ to 70%^33^ of $ET$ in areas with adequate rainfall or irrigation. By scaling this ratio to the converted land areas from those four countries, converting mangrove to oil palm has potentially led to an additional 21.6 to 58.4 GL H_2_O year^−1^ toward transpirational water loss that would simultaneously reduce water flows toward estuaries. As oil palm plantations often need irrigation in dry years, the additional amount of freshwater needed to sustain oil palm ${E}_{c}$ becomes unavailable for human use and to support ecological processes during irrigation years, when societal demands are likely to be highest^34^. Furthermore, freshwater use by mangroves would not compete directly with humans under most land use or climate change scenarios.

If there are large carbon costs of water uptake and transport for mangroves growing in saline intertidal environments, then maintaining plant growth must be balanced by low water costs of carbon gain at some organizational scale^19^. Initial water use studies that measured sap flow in mangroves actually found appreciable rates of water flux in the outer sapwood of trees. This result suggested that the velocity of xylem water ascent in mangroves may not be distinctively low, despite the environmental conditions under which mangroves develop. For example, median sap velocities of mangroves from outer sapwood locations averaged ~ 0.13 mm s^−1^ in Borneo and Hawaii, USA^35,36^, which did not differentiate strongly from median velocities of ~ 0.13–0.16 mm s^−1^ in adjacent upland tropical dipterocarp or heath forest trees^35^. However, volume of water flux in mangroves is low versus many terrestrial forest trees (Fig. 1b) because sap flow with depth into sapwood (from cambium beyond depths of 2 cm) attenuates quickly^37,38^, which reduces the total amount of water ascending the tree stem to become available to foliage. Canopy leaf-area is adjusted in sync with stem capacity for water transport; with canopies dying back when water is less available or expanding as water supply increases^39,40^. This suggests that limited water use in mangroves may be a function of stem adjustments in water transport with changing environmental conditions, which may be imposed in concert with leaf-scale response.

**Figure 3:**
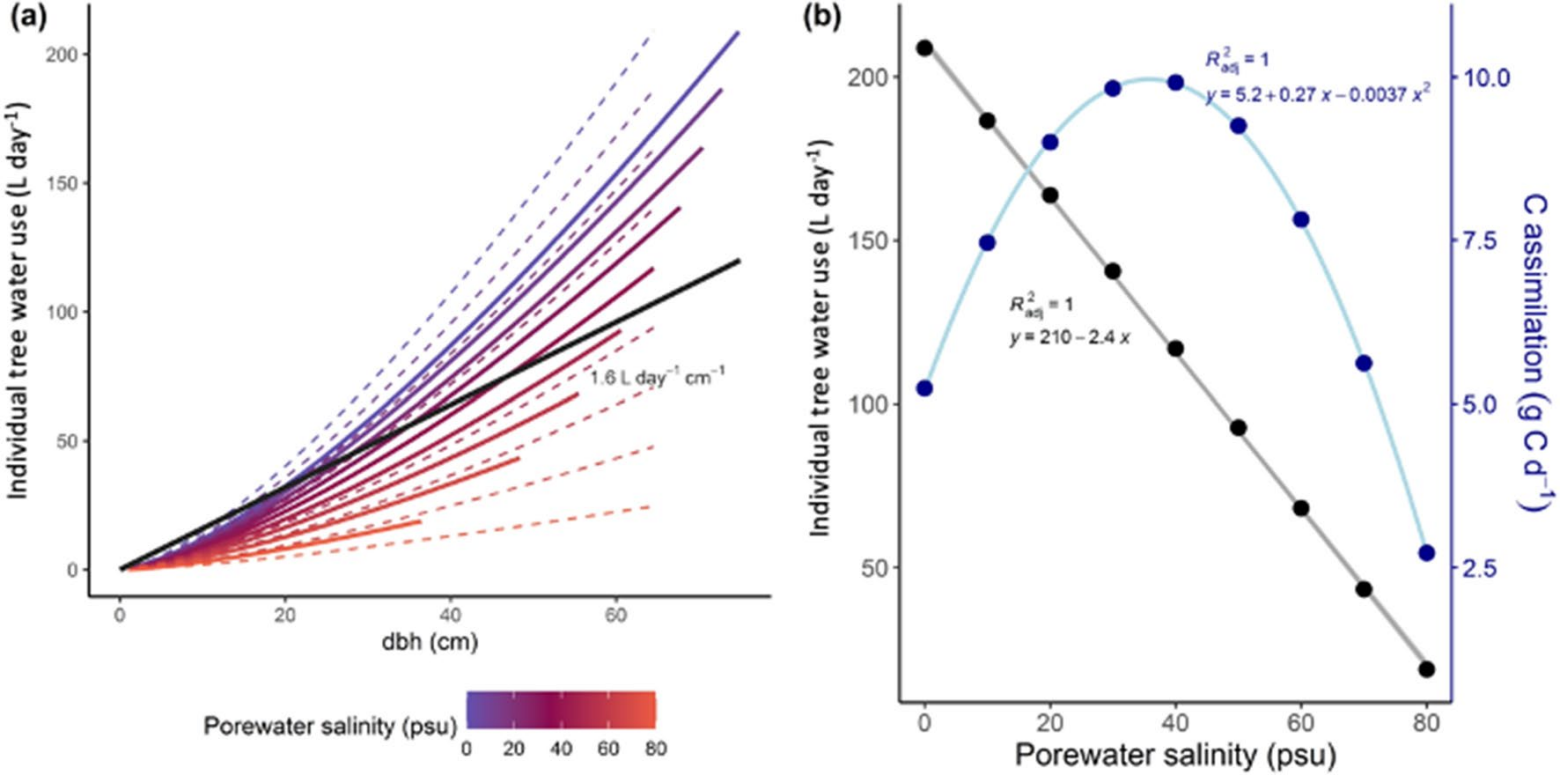
BETTINA model simulations applied to tree size and salinity vs. water use. (**a**) Individual tree water use from BETTINA modelling studies relative to dbh for different salinities within mangroves. Each solid line represents one tree over the time of simulation with trees adapting their allometry to variation in soil salinity (from blue for 0 psu to red for 80 psu). For comparison, dashed lines mark the water uptake at different salinities with no allometric adaptation (average tree allometry as growing at 40 psu); solid black line represents empirical individual tree water use estimated for mangroves in this study. (**b**) Simulated individual tree water use of each mangrove tree at an age of 200 years.

To expand on this idea mechanistically, we applied the BETTINA Model^41^, which was previously parameterized for a widely adapted and globally distributed mangrove genus, *Avicennia*^42^. Simulations provide theoretical estimations of individual tree water use versus tree size and salinity given adequate water and light resources (Fig. 3). Depending on the salinity, trees exhibit differential biomass allocation patterns and biomass structural differences that influence water use. In fact, modelled trees become shorter but have a proportionally larger stem diameter with increasing salinity (Fig. 3a), which is in good agreement with field data^43^. The structural adjustments under saline conditions result in hydraulic architecture (e.g., modified canopy area) that ensure optimal water potentials and sap flow with increasing environmental stress. Thus, in addition to inherently lower whole-tree water use in mangroves compared to upland trees, saline conditions exert additional constraints to whole-tree mangrove water use. For example, water use can become theoretically high (> 150 L H_2_O day ^−1^), and far greater than the literature has documented to date (Fig. 3b), when mangrove trees are large and without major constraints on water use imposed by high salinity; also affecting actual-to-potential transpiration (Fig. S3). For modelled trees, the water use of a tree growing at 70 psu (double the salt concentration of seawater) is 21% the water use of a tree growing with zero salinity (Fig. 3b).

Plasticity in water use along environmental gradients has also been studied from anatomical and physiological perspectives in mangroves^44^. However, this topic remains little explored at the whole-tree architectural level. Past studies reveal two important considerations. First, mangrove tree species growing under increasingly saline environments can have greater xylem vessel grouping and vessel density, as well as reduced vessel diameters and lengths than their low-salinity conspecifics, which is proposed to reduce the risk of embolisms associated with highly negative water potentials^45^. Second, some mangroves – those that secrete salt from their leaf and bark surfaces – can dynamically control xylem ion composition^46^ to increase hydraulic conductivity^47^ as water potential becomes more negative, whilst maintaining relatively stable stomatal conductance (and transpiration). This dynamic xylem sap osmolality control increases the water available to the transpiration stream without actively increasing transpiration, further reducing the risk of embolisms under salt stress^46^.

**Figure 4:**
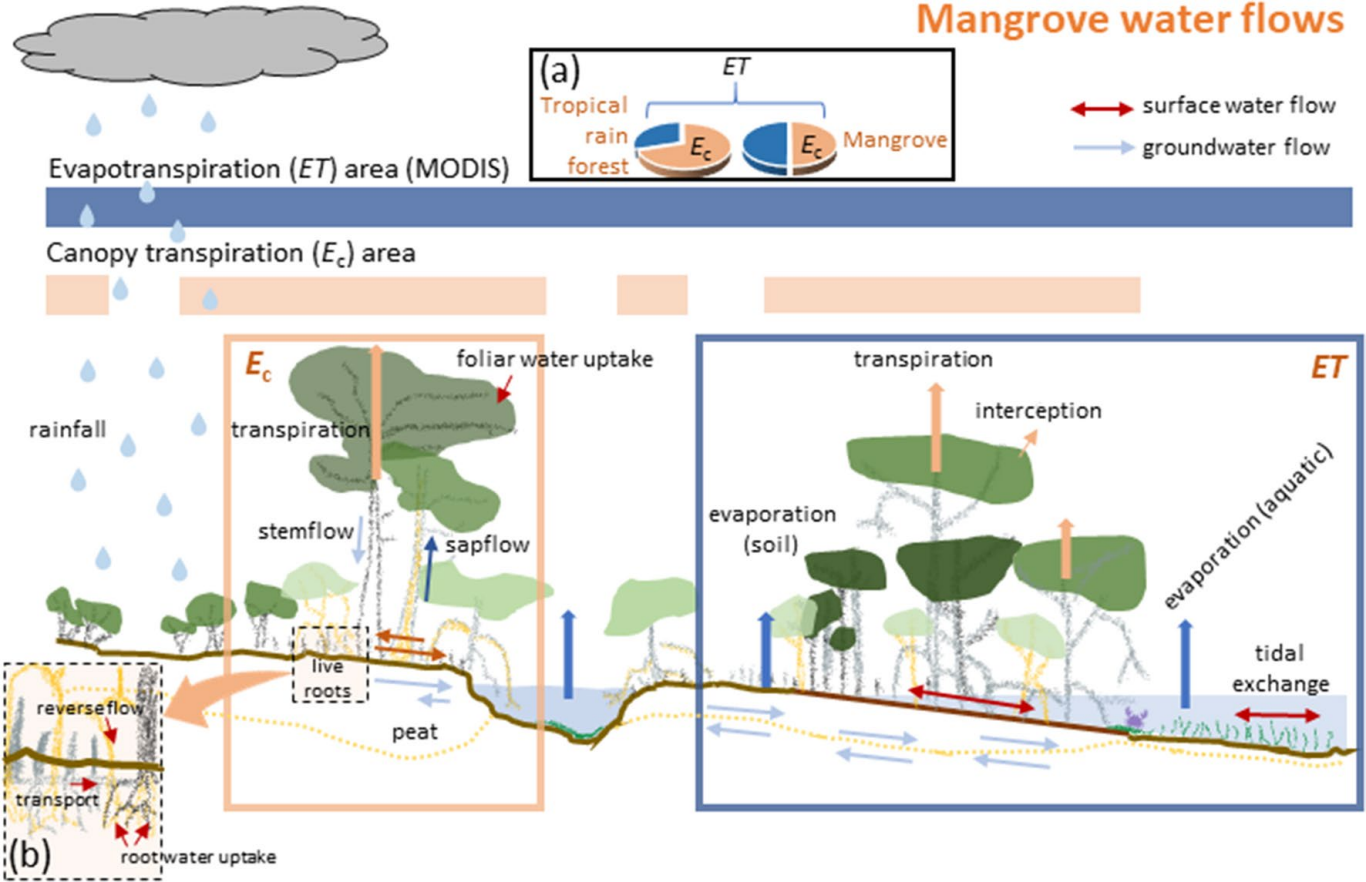
Water flows in mangrove forests associated with either evapotranspiration (*ET*) or canopy transpiration (*E*_c_), and how areas from MODIS-derived *ET* data and *E*_c_ data would relate for mangroves, as well as generally from the literature for other ecosystems. (**a**) Inset represents a comparison of ${E}_{c}$-to-$ET$ ratio for mangroves versus tropical rain forests, where most of the world’s mangroves associate. (**b**) Inset represents known water fluxes in mangrove root zones that contribute to *E*_c_, and ultimately to *ET*.

For our analysis focused at the broader canopy scale, we report average ${E}_{c}$ alongside of location-specific $ET$ at the scale of 1-km^2^ from the MODIS satellite product (MOD16-A3), which is an analytical approach used by Schlesinger & Jasechko^48^ to permit direct comparisons of sap flow-derived transpiration rates and $ET$ among a suite of different ecoregional vegetation types. It is important to recognize that this approach provides an approximation of ${E}_{c}$ from theoretically continuous vegetation coverage versus MODIS-derived $ET$ over the same ground area; i.e., MODIS would also include areas without mangroves (Fig. 4). MODIS-derived $ET$ includes several non-${E}_{c}$ mechanisms of water loss as well as sub-areas not continuously occupied by vegetation, so we considered water use for mangroves compared to non-mangrove ecoregions using ${E}_{c}$-to-$ET$ ratios to better control for different, but inherent, $ET$ water fluxes from non-transpirational sources.

At least four assumptions apply to this ${E}_{c}$-to-$ET$ ratio analysis. First, NPP data from each mangrove wetland location, and thus ${E}_{c}$ approximations, are from the study plots used in the cited studies, and therefore, may not represent the average regional structure of the entire mangrove forest over 1 km^2^. We assume that plots are representative of the larger stand. Second, our approximations of ${E}_{c}$ focus just on the dominant vegetation and do not include evaporation from flooded soil, saturated soil, tidal creeks, and leaf-intercepted water, or the tide-energy influences on water losses, which would be part of what $ET$ would include^49^. Third, our sap flow generated determinations of ${E}_{c}$ may exclude some components of the understory. However, the degree to which source-study NPP measurements included saplings and small trees of the understory would influence our estimates of ${E}_{c}$; the more inclusive of those understory NPP components from the original data sources, the better our ${E}_{c}$ approximations. Finally, for the salinity component specific to mangroves, while freshening of water has the potential to increase ${E}_{c}$-to-$ET$ ratios, we assume that $ET$ across larger spatial scales is altered only modestly with salinity reduction. To our knowledge, salinity-to-$ET$ relationships have not been assessed at relevant scales to consider further.

Empirical assessment of ${E}_{c}$-to-$ET$ ratios is limited to only a few mangrove studies. On sites in Southwest Florida, USA with moderate stand basal areas (23–28 m^2^ ha^−1^) and average canopy heights (10–11 m), regional $ET$ was 1029–1048 mm H_2_O year^−1^ while dominant canopy ${E}_{c}$ was 350–511 mm H_2_O year^−1^, or 34–50% of $ET$ ^50^. Short mangrove forests (< 5 m tall) on Hainan Island, China, used only 269–357 mm H_2_O year^−1^ versus an $ET$ of 1134–1166 mm H_2_O year^−1^, or 23–31% of $ET$ ^51^. Mangrove stands did use as much as 872 mm H_2_O year^−1^ versus an $ET$ rate of 1378 mm year^−1^ (or 63% of $ET$) in an Everglades mangrove stand, also in south Florida, but in a location were the stand had moderate salinity concentrations (~ 25 psu), greater basal area (41 m^2^ ha^−1^), and taller trees (19 m)^50^. ${E}_{c}$-to-$ET$ ratios ranged from 30 to 66% among three additional mangrove sites in China, with these ratios linked to seasonal leaf area index and salinity change^52^. Unlike the direct linear increases in ${WUE}_{int}$ described at the leaf-level as salinity increased (Fig. S1)^20^, mangrove ${E}_{c}$ remained fairly constant over salinities of ~ 10 to 28 psu and decreased beyond^52^. Thus, empirical estimations of ${E}_{c}$-to-$ET$ ratios fall within the range of our derived values. In fact, any discrepancies trended toward more water-use conservative ${E}_{c}$-to-$ET$ ratios for mangrove vegetation.

By aligning global mangrove area with the distribution of adjacent ecoregions to mangroves, water use differences from our analysis scale to 34.7 Teraliters (TL) H_2_O year^−1^ if comparable areas to mangrove are assumed (Table 1, Table S1). We add this perspective to demonstrate that having mangroves along a coast can indeed be consequential to water cycling locally as well as globally. Even under an assumption of low soil porewater salinity, consumptive water use by mangroves is still comparatively low (by 16.2 TL H_2_O year^−1^). Likewise, our predictive variable (NPP) for approximating ${E}_{c}$ among the world’s mangroves is linked strongly to rainfall, explaining up to 86% of global carbon stocks among mangroves when compared with other climatic variables, such as atmospheric temperature^53^. Thus, mangroves receiving higher rainfall benefit from reduced salinities and increased nutrients, which in turn illicit productivity gains requiring more freshwater use by trees.

Finally, there is a growing concern for the ability to feed the Planet’s increasing population with limited renewable freshwater resources^27,54^, due to changes in diets, increased demand for freshwater resources to support fossil fuel extraction^55^, and competition between water used for agricultural production of biofuels and food^56^. Placing potential water use reductions by mangroves within this food-energy-water nexus requires understanding of the multiple ecosystem services that mangrove wetlands provide, which encompass food resources, building materials and energy utilization. The quantification and comparison of freshwater consumption in the production of 268 goods and services can be determined from estimates of ${E}_{c}$ by attributing water use per hectare to consumable weights (Mg) of resultant products in marine and blue carbon ecosystems. Water used in the production of a consumable product accompanies that product as a legacy requirement during local or global trade as the product realizes its destination and ultimate use; this water is defined as “virtual water content”^57^.

When we evaluate virtual water content from mangroves versus relevant ecoregions based on global estimates of the economic value of ecosystem services for each^58^, we estimate that the value of food production per hectare for a tropical rainforest (e.g.), in USD, is approximately $ 0.27 mm^−1^ of H_2_O but for mangroves it is $ 2.20 mm^−1^ of H_2_O (Table S2). Similarly, the raw material value is $ 0.11 mm^−1^ of H_2_O for tropical rainforests compared to six-fold higher value for mangroves. Over time, such analysis may reveal previously unrealized returns on investments in protection and restoration of mangroves, and possibly of other emergent blue carbon wetlands.

## Conclusions

Efficient and conservative use of freshwater at the individual tree and stand levels by mangroves equate to global water use differentials in the trillions of liters annually compared to adjacent ecoregions and alternate land use area. We can gain additional information on levels of water use from other blue carbon wetlands through expanding eco-hydrologic studies, especially as these wetlands may also be efficient in maintaining their functions with reduced freshwater availability. Low freshwater use by mangroves could significantly augment the global blue carbon wealth of nations, recently estimated to be $190.67 ± 30 billion year^−1^ (USD)^59^. High productivity of mangroves for their low usage of freshwater compared to land uses that have replaced them provides evidence of additional benefits to the ecology of associated estuaries that are dependent on freshwater flows. The water economy of ecosystems may require greater worldwide consideration as humans look for options to ameliorate, limit, and adapt to future water shortages while sustaining coastal productivity and ecological connections. The conservative water use of blue carbon ecosystems may add to their value and function as nature-based solutions, and to coastal resilience as freshwater availability is reduced.

## Materials and methods

At least 11 coastal ecosystems have been considered based on a minimum of actionably defined criteria to be “blue carbon ecosystems”. These include mangrove wetlands, tidal marshes (salt, brackish, fresh), seagrasses, salt flats, freshwater (upper estuarine) tidal forests, macroalgae, phytoplankton, coral reef, marine fauna (fish), oyster reefs, and mud flats^5^; all but three of these would be considered wetlands, with salt flats and mud flats being examples of non-emergent (plant) blue carbon wetlands. Herein, we focus on mangroves.

### Adjusting intrinsic leaf-level photosynthetic water use efficiency (${WUE}_{int}$) in response to environmental gradients (Introduction)

We used data provided by B.F. Clough & R.G. Sims^20^, which presented leaf-scale net photosynthesis (${P}_{n}$ [sic]; μmol CO_2_ m^−2^ s^−1^), stomatal conductance (${g}_{w}$: mol m^−2^ s^−1^), leaf-intercellular CO_2_ concentrations (${c}_{i}$: μl l^−1^), and intrinsic photosynthetic water use efficiency (${WUE}_{int}$: $\frac{{P}_{n}}{{g}_{w}}$) for 19 mangrove species occupying 9 different sites in Papua New Guinea and northern Australia. These field data were collected using an infrared gas analyzer (model Li-6000, Li-Cor Biosciences, Inc., Lincoln, NE, USA) attached to leaves at saturation light levels (reported as > 800 μmol PPFD m^−2^ s^−1^). Soil salinity at the time of data collection ranged from 10 to 49 psu, and median long-term atmospheric temperature and relative humidity among sites ranged from 19.9 to 27.4 °C and 35.1 to 92.2%, respectively (Fig. S1)^20^. These data were among the first to offer insight from field study into the plasticity of mangroves across a range of natural salinity and aridity gradients to adjust leaf-level ${WUE}_{int}$ as needed for local environmental condition. While it is not new for trees to adjust their ${WUE}_{int}$ when they develop in arid, semi-arid, or even some humid and tropical environments^60^, what is distinctive is that mangroves may be further driven to water savings by salinity gradients as a condition of development.

### ${{\varvec{W}}{\varvec{U}}{\varvec{E}}}_{{\varvec{i}}{\varvec{n}}{\varvec{t}}}$ and individual tree water use of mangrove wetlands versus terrestrial ecosystems

For Fig. 1a, we compare leaf-level ${WUE}_{int}$ data collected from 17 published papers (using maximum and minimum values), providing 67 independent measurements of ${WUE}_{int}$ for mangroves (Table S3). While we mention in the main text that as many as 214 independent measurements of water use efficiency are available, not all of these present raw ${P}_{n}$ or ${g}_{w}$ data, with some reporting leaf transpiration (${T}_{r}$) which do not enable reporting of intrinsic water use efficiencies. Also, we strategically included studies from reproducible experimental designs and readily available papers. Mangrove species included in this review represented a global distribution of greenhouse and field observations, and encompassed species in the following mangrove genera: *Rhizophora*, *Avicennia*, *Laguncularia*, *Bruguiera*, *Aegialitis*, *Aegiceras*, *Ceriops*, *Sonneratia*, *Kandelia*, *Excoecaria*, *Heritiera*, *Xylocarpus*, and *Conocarpus*.

We then accessed an existing database (n = 11,328 observations) that reported raw ${P}_{n}$ and ${g}_{w}$ data from 210 upland deciduous and evergreen shrubs and trees of savannah, boreal, temperate, and tropical habitats^60^. From these data, we evaluated a range of upland tree and shrub species that occurred and developed naturally in environments along a global gradient of vapor pressure deficit (i.e., atmospheric moisture and temperature), including arid, semi-arid, dry semi-humid, and humid locations.

For Fig. 1b, we started with a review by Wullschleger et al.^61^ that provides maximum individual tree water use data (L H_2_O day^−1^) from 52 published studies representing 67 species of upland trees from around the world. Of those studies, *dbh* values (8 to 134 cm) were provided alongside 47 individual tree water use values. Maximum individual tree water use and *dbh* (4.1 to 45.3 cm) were available from the original source for 8 mangrove studies representing 7 species from French Guiana, Mayotte Island (Indian Ocean), China, Florida (USA), and Louisiana (USA) (Table S4). These represent the extent of published sap flow data that provided both individual tree water use and *dbh* from mangroves (numerically); e.g., we could not extract specific individual tree water use versus *dbh* from a Moreton Bay (Australia) study site^62^, south Florida study site^63^, or from five additional study sites in China^51,52^. However, regressions for two of the Chinese study sites provided over two years^51^ indicated that mangrove trees from a suite of species ranging in *dbh* from 8 to 24 cm used approximately 0.76 and 9.31 L H_2_O day^−1^, or 0.53 L H_2_O day^−1^ cm^−1^ of *dbh*. These apparent rates were even lower than what was reported as average for mangroves in Fig. 1b of 1.4 L H_2_O day^−1^ cm^−1^. The mangrove species included in this analysis were *Avicennia germinans* (L.) L., *Laguncularia racemosa* (L.) C.F. Gaertn., *Rhizophora mangle* L., *Ceriops tagal* (Perr.) C.B. Rob., *Rhizophora mucronata* Lam., *Sonneratia apetala* Buch.-Ham, and *Sonneratia caseolaris* (L.) Engl.. Additional comparative mangrove species reported by B. Leng & K.-F. Cao^51^ included *Bruguiera sexangula* (Lour.) Poir., *Bruguiera sexangula* var. *rhynchopetala* W.C. Ko, *Excoecaria agallocha* L., *Rhizophora apiculata* Blume, *Sonneratia alba* Sm*.*, and *Xylocarpus granatum* J. Koenig.

### Estimation of canopy transpiration (${{\varvec{E}}}_{{\varvec{c}}}$) from net primary productivity data

Estimates of carbon uptake from CO_2_ can provide insight into the water use requirement for that uptake of carbon^64^. We used leaf-level instantaneous water use efficiency (${WUE}_{ins}$: $\frac{{P}_{N}}{{T}_{r}})$, which relates to net CO_2_ uptake from leaves of the dominant mangrove forest canopy relative to the specific amount of water used, and developed a predictive relationship (*predicted*) for determining mangrove net primary productivity (NPP) values from ${E}_{c}$ using ${WUE}_{ins}$. For *A. germinans*, *L. racemosa*, and *R. mangle* forest components, we used light-saturated, leaf-level ${WUE}_{ins}$ values of 3.82 ± 0.3, 4.57 ± 0.3, and 5.15 ± 0.4 mmol CO_2_ (mol H_2_O)^−1^ [± 1 SE], respectively, from mangrove saplings and small trees of south Florida^65^. ${WUE}_{ins}$ values were stratified by species relative to basal area distributions on each south Florida study plot, converted from molar fractions of H_2_0 (from ${E}_{c}$ determination) and CO_2_ to molecular weights, and multiplied by ${WUE}_{ins}$ with applicable unit conversions to attain kg CO_2_ m^−2^ year^−1^. This value was multiplied by 0.273 to yield kg C m^−2^ year^−1^.

This predictive relationship was validated in two independent ways. First, for one of the calibration sites (lower Shark River, Everglades National Park, Florida, USA), we modeled ${E}_{c}$ from sap flow data^50^, determined NPP from ${WUE}_{ins}$ calculations relative to the amount of water the stand used, and had independent measurements of net ecosystem exchange (NEE) of CO_2_ between the mangrove ecosystem and atmosphere from an eddy flux tower^66^. For this site, NPP estimation and NEE were closely aligned once soil CO_2_ effluxes were accounted; respiratory CO_2_ effluxes from soil and pneumatophores were determined to be 1.2 kg C m^−2^ year^−1^ from previous study^67^. Using our NPP estimations from ${WUE}_{ins}$ calculations and subtracting soil and pneumatophore CO_2_ effluxes of 1.2 kg C m^−2^ for 2004 and 0.8 kg C m^−2^ for 2005 (partial year), NPP becomes 0.96 kg C m^−2^ for 2004 and 0.85 kg C m^−2^ for January to August of 2005 (see *Observed 1, Florida* on Fig. S2). Our approach underestimated NPP from ${E}_{c}$ relative to measurements from eddy covariance by 0.21 kg C m^-2^ for 2004 (within 17.5% of *predicted*) and was nearly identical for 2005 (within 0.02 kg C m^−2^, or 2% of *predicted*).

Second, we wanted to determine whether ${E}_{c}$-to-NPP predictions developed on a few sites in south Florida, USA, represented other global sites, so we included an analysis from several mangrove sites in Guangdong Province, China, to represent an entirely different location. Similar to south Florida analyses, we combined data for NPP from co-located sites of ${E}_{c}$ determination using sap flow techniques. NPP of the mangrove forests were measured using multiple procedures (including eddy flux) for improved accuracy^68,69^. The relationships of predicted NPP versus ${E}_{c}$ and observed NPP versus ${E}_{c}$ did not differ between Florida and China (t = 0.48, *p* = 0.643).

### Projecting mangrove ${{\varvec{E}}}_{{\varvec{c}}}$ to other locations

We reviewed data from 26 published records that report mangrove NPP, or enough data to estimate NPP, from 71 study sites located in the Florida-Caribbean Region (25 sites) and Asia–Pacific Region (46 sites) (Table S5). Table S1 reveals mangrove literature sources used, as well as how NPP was estimated from values provided in the original source; itemizes assumptions for determinations of aboveground NPP, wood production, litter production, and root production from various ratios^70^; and reveals unit conversions.

We then convert NPP to ${E}_{c}$ for all 71 sites using the *predicted* curve in Fig. S2 (Eq. 1, main text), and provide summary results by location in Table S1. Regional $ET$ data were extracted from the MODIS Global Evapotranspiration Project (MOD16-A3), which are provided at a resolution of 1-km. The locations of mangrove NPP study sites were identified, assigned to a single 1-km^2^ grid in MOD16, and $ET$ was extracted from that grid and used for ${E}_{c}$-to-$ET$ comparison. Average $ET$ from single cells (1 km^2^) was combined with the average of up to 8 additional neighboring cells to provide comparative $ET$ projections over up to 9 km^2^ for each location from 2000 to 2013 to compare sensitivity among suites of the specific MODIS16-A3 cells selected over land. When neighboring cells were completely over water, they were excluded since component mangrove forest ${E}_{c}$ estimation was not possible from the cells. Estimates of $ET$ by individual cells used to compare with mangrove ${E}_{c}$ versus up to 9 cells differed by an average of only 43 mm H_2_O year^−1^ (± 16 mm H_2_O year^−1^, S.E.). Therefore, we use $ET$ from individual, overlapping ${E}_{c}$ cells in Table S1.

The average ${E}_{c}$-to-$ET$ ratio from mangroves was subtracted from ${E}_{c}$-to-$ET$ ratio for specific ecoregions^48^, and this ratio difference was assumed to represent net water use strategy affecting differences by the dominant vegetation between ecosystem types. We were also mindful that salinity reductions can affect ${E}_{c}$. We used scaled (0–1) mean and standard deviations from ${WUE}_{int}$ data previously reported for mangroves (Fig. S1)^20^. Standard deviation was 32% of mean ${WUE}_{int}$ related to salinity gradients, and if we re-scale this deviation to ${E}_{c}$ data and add it to the mean ${E}_{c}$ to assume low salinity, average ${E}_{c}$-to-$ET$ ratio becomes 57.4%. This is theoretical and assumes a relatively linear relationship between ${WUE}_{int}$ and ${E}_{c}$.

### Comparative water use scaling among ecoregions

Table 1 presents the projected reduction in water used through ${E}_{c}$ if a mangrove ${E}_{c}$-to-$ET$ ratio was applied to tropical rainforest (290.52 mm H_2_O year^−1^), temperate deciduous forest (131.76 mm H_2_O year^−1^), tropical grassland (110.77 mm H_2_O year^−1^), temperate grassland (46.48 mm H_2_O year^−1^), temperate coniferous forest (54.96 mm H_2_O year^−1^), desert (22.99 mm H_2_O year^−1^), and Mediterranean shrubland (12.08 mm H_2_O year^−1^). To convert potential water use differences to kL H_2_O ha^−1^ year^−1^ (as presented in the abstract), the following calculation is used (using the example of tropical rainforest):

2 $$\frac{290.52 L {H}_{2}O {year}^{-1}}{1 {m}^{2}} \times \frac{\mathrm{10,000 }{m}^{2}}{1 ha} \times \frac{1 kL {H}_{2}O}{\mathrm{1000 }L {H}_{2}O} =\mathrm{2905 } kL {H}_{2}O {ha}^{-1}{year}^{-1}$$

For comparisons made to mature (> 12 years) oil palm (*Elaeis guineensis* Jacq.) plantations, ${E}_{c}$-to-$ET$ ratio was assumed to range from 53^32^ to 70%^33^, for a water use difference of 1170 and 3160 kL H_2_O ha^−1^ year^−1^, respectively, relative to annual global mangrove $ET$ (of 1172 mm). We multiply these values by the 18,467 ha of land area that was converted from mangroves to oil palm^31^ to attain potential water use differences of 21.6 to 58.4 GL H_2_O year^−1^ from avoided conversion of mangrove to oil palm in this region.

### Global water use scaling

In order to determine how much global mangrove area is adjacent to each ecoregion, we conducted a cross-walk between terrestrial ecoregions^71^ and those used by Global Mangrove Watch in the 2010 classification of global mangrove area^72^. Terrestrial ecoregions used by Schlesinger & Jasechko^48^ were then able to be associated with specific mangrove areas (Table S6). In other words, given a specific ecoregion, we determined how much mangrove area would be occurring within that same ecoregional geography. Global mangrove area assignment to those ecoregions mapped within 0.1% of the total mangrove area of 13,760,000 ha reported in Bunting et al.^72^. To convert kL H_2_O ha^−1^ year^−1^ to GL H_2_O year^−1^ among ecoregions, the following calculation was used (continuing with the example of tropical rainforest, which has an area of adjacent mangroves of 112,331.9 km^2^):

3 $$\frac{\mathrm{2905} kL {H}_{2}O {year}^{-1}}{1 ha} \times \frac{100 ha}{1 {km}^{2}} \times \frac{\mathrm{112,331.90} {km}^{2 }mangroves}{1.0 \times {10}^{6} kL {H}_{2}0} \times \frac{1 GL {H}_{2}O}{1} = \mathrm{32,632.42} GL {H}_{2}O {year}^{-1}$$

### Agent-based modelling of individual tree water use (Discussion)

The BETTINA model simulates the growth of mangrove trees as a response to above- and below-ground resources, i.e. light and water^41^. In the model, an individual tree is described by four geometric measures, including stem radius, stem height, crown radius and root radius; attributing functional relevance in terms of resource uptake. Aiming to maximize resource uptake, new biomass is allocated to increase these measures in an optimal but not constant proportion. Water uptake of the tree is driven by the water potential gradient between the soil and the leaves. Thus, porewater salinity is part of what determines the water availability for plants.

With the BETTINA model, we simulated the growth of nine individual mangrove trees under different salinity conditions, ranging between 0 and 80 psu, while all other environmental and tree-specific conditions were kept constant. Simulation time was 200 years so that trees could achieve very close to their maximum possible size, and the hydrological parameters were similar to that reported previously^42^. We can show that the ratio of the actual transpiration to the potential transpiration decreases with increasing salinity; plants use less water. Potential transpiration was the transpiration of a given tree without a simulated reduction in water availability due to porewater salinity. These parameter details are presented graphically for mangroves (Fig. S3), comparing porewater salinity along a gradient against the ratio of actual-to-potential individual tree transpiration.

Further, BETTINA simulation results include morphological plasticity adjustments to allometry. To highlight this, we also displayed results assuming a constant allometry as for 40 psu. Naturally, for this arbitrary benchmark the solid and the dashed line coincide (Fig. 3a). Adaptation to higher salinities improves water uptake (primarily girth and root growth), thus the adapted trees (solid lines) have a higher water uptake than the average allometry (dashed lines) for salinities below 40 psu. Lower salinities promote increase of height and crown radius to improve light availability. That is why the adapted trees have a lower water uptake than an average tree would for salinities above 40 psu. Tree water use decreases with increasing salinity (Fig. 3b), as ${WUE}_{int}$ coincidently increases (Fig. S1).

### Virtual water use explained (Discussion)

Water is required to produce products or acquire services from natural ecosystems; e.g., forest products, fisheries biomass, nutrient processing (nitrification, denitrification), food production. If a net kilogram of a food is grown on a hectare of land where water is abundant and that kilogram of food requires 400 mm of water to be produced, the export of that food to an area of low water availability provides an ecosystem service in the amount of 1 kg of food, plus 400 mm of “virtual” water not actually needed at the destination but used at the source. This water is defined as the product’s “virtual water content”^56^. There is a rich body of literature exploring the concept of virtual water^73,74^, but we expand on this concept here as a comparison among 7 ecoregions^48^ and mangroves. Raw data used for calculations are presented in Table S2.

### Statistical analysis

Data for leaf-level ${WUE}_{int}$ comparisons between terrestrial woody plants and mangroves, as well as individual tree water use by *dbh* for both terrestrial and mangrove trees, were not normally distributed. We used a Kruskal–Wallis ANOVA based on ranks, and the Dunn’s Method for difference tests. Individual tree water use by *dbh* for both terrestrial and mangrove trees were determined using linear regression, mostly applied to mean values. For a couple of mangrove studies, only median values were extractable from minimum and maximum values. Likewise, all other data relationships were best fit with linear models, including the calibration curves between ${E}_{c}$ and NPP. All data were analyzed using SigmaPlot (v. 14.0, Systat, Inc., Palo Alto, California, USA).

## Supplementary Information

Supplementary Information.

## Data Availability

All data generated or analyzed during this study are included in this published article [and its supplementary information files], or from https://bitbucket.org/gsglobal/leafgasexchange for data reported in Medlyn et al.^60^. Model code for the BETTINA model (a sub-routine of the model, MANGA) is available at, https://github.com/mcwimm/bc_wetlands.
