# Supplementary material for: Mangroves provide blue carbon ecological value at a low freshwater cost
Source: Sci Rep. 2022 Oct 21;12:17636. doi: 10.1038/s41598-022-21514-8 (PMC9586979; doi:10.1038/s41598-022-21514-8)
Supplement: Supplementary file 1 — Supplementary Information. [file 41598_2022_21514_MOESM1_ESM.docx]

SUPPLEMENTARY INFORMATION

**Mangroves provide blue carbon ecological value at a low freshwater cost**

Ken W. Krauss,^1,*^ Catherine E. Lovelock,^2^ Luzhen Chen,^3^ Uta Berger,^4^ Marilyn C. Ball,^5^ Ruth Reef,^6^ Ronny Peters,^4^ Hannah Bowen,^7^ Alejandra G. Vovides,^8^ Eric J. Ward,^1^ Marie-Christin Wimmler,^4^ Joel Carr^9^, Pete Bunting^10^, Jamie A. Duberstein^11^

^1^U.S. Geological Survey, Wetland and Aquatic Research Center, Lafayette, LA 70506, USA. ^2^School of Biological Sciences, The University of Queensland, Brisbane 4072, Australia. ^3^Key Laboratory of the Ministry of Education for Coastal and Wetland Ecosystems, College of the Environment and Ecology, Xiamen University, Xiamen, Fujian 361102, China. ^4^Institute of Forest Growth and Forest Computer Sciences, Technische Universität Dresden, 01062 Dresden, Germany. ^5^Research School of Biology, The Australian National University, Acton ACT 2601, Australia. ^6^School of Earth, Atmosphere and Environment, Monash University, Clayton VIC 3800, Australia. ^7^Instituto de Ecología AC, Carretera antigua a Coatepec 351, Xalapa 91073 Veracruz, México. ^8^School of Geographical and Earth Sciences, University of Glasgow, Glasgow, U.K. ^9^U.S. Geological Survey, Eastern Ecological Science Center, Laurel, MD 20708, USA. ^10^Department of Geography and Earth Sciences, Aberystwyth University, Aberystwyth, Wales, U.K. ^11^Baruch Institute of Coastal Ecology and Forest Science, Clemson University, Georgetown, SC 29442, USA. *e-mail: [**kraussk@usgs.gov**](mailto:kraussk@usgs.gov)


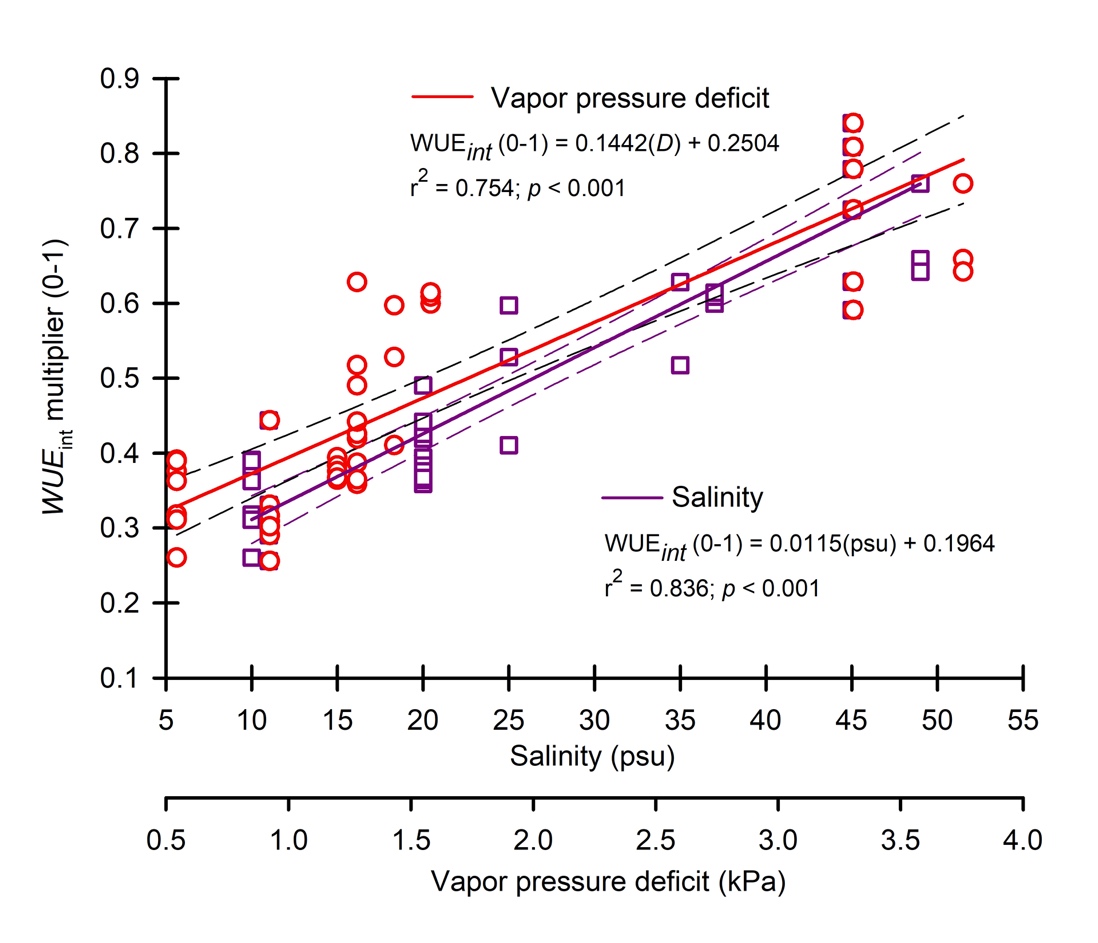


**Figure S1. Multiplier-scaled (0-1) response of leaf-intrinsic water use efficiency (**$\boldsymbol{WUE}_{\boldsymbol{int}}$**) versus salinity and atmospheric vapor pressure deficit.** These data were collected for a suite of 19 mangrove species occupying 9 different mangrove field sites in Papua New Guinea and northern Australia^20^. A value of 1 on the *y*-axis equates to a ${WUE}_{int}$ of 121.7 μmol CO_2_ (mol H_2_O)^-1^.


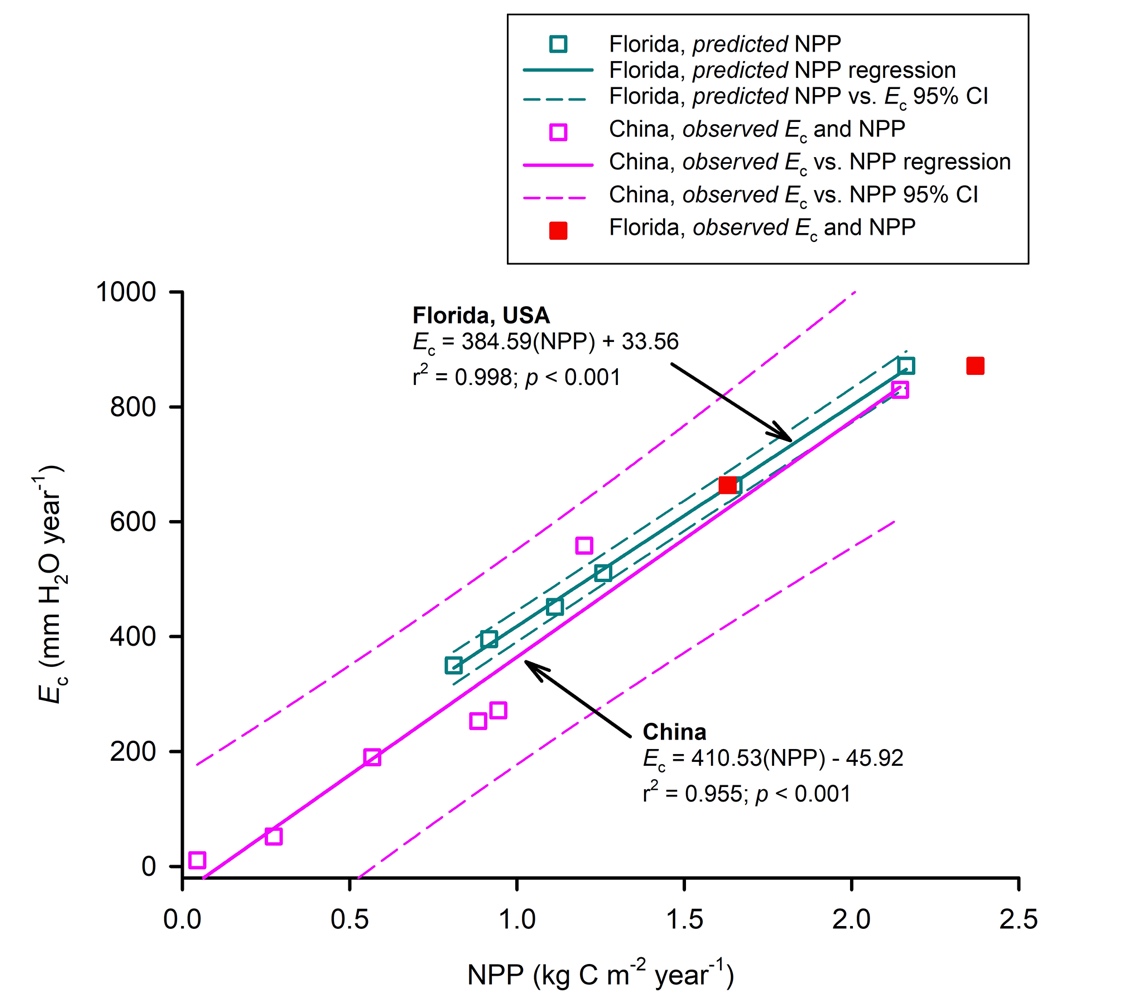


**Figure S2. Projected rates of dominant canopy transpiration (**$\boldsymbol{E}_{\boldsymbol{c}}$**) from net primary productivity (NPP, kg C m^-2^ year^-1^).** For data used in this analysis (blue-green squares), we include the 95% confidence interval (*predicted*), versus empirical measurements of NPP and $E_{c}$ over 2 time periods on one site along the lower Shark River (*observed 1, Florida* – solid red squares) and from empirical measurements of NPP and $E_{c}$ over single time periods from 7 sites in Guangdong Province, China (*observed 2, China* - pink squares).


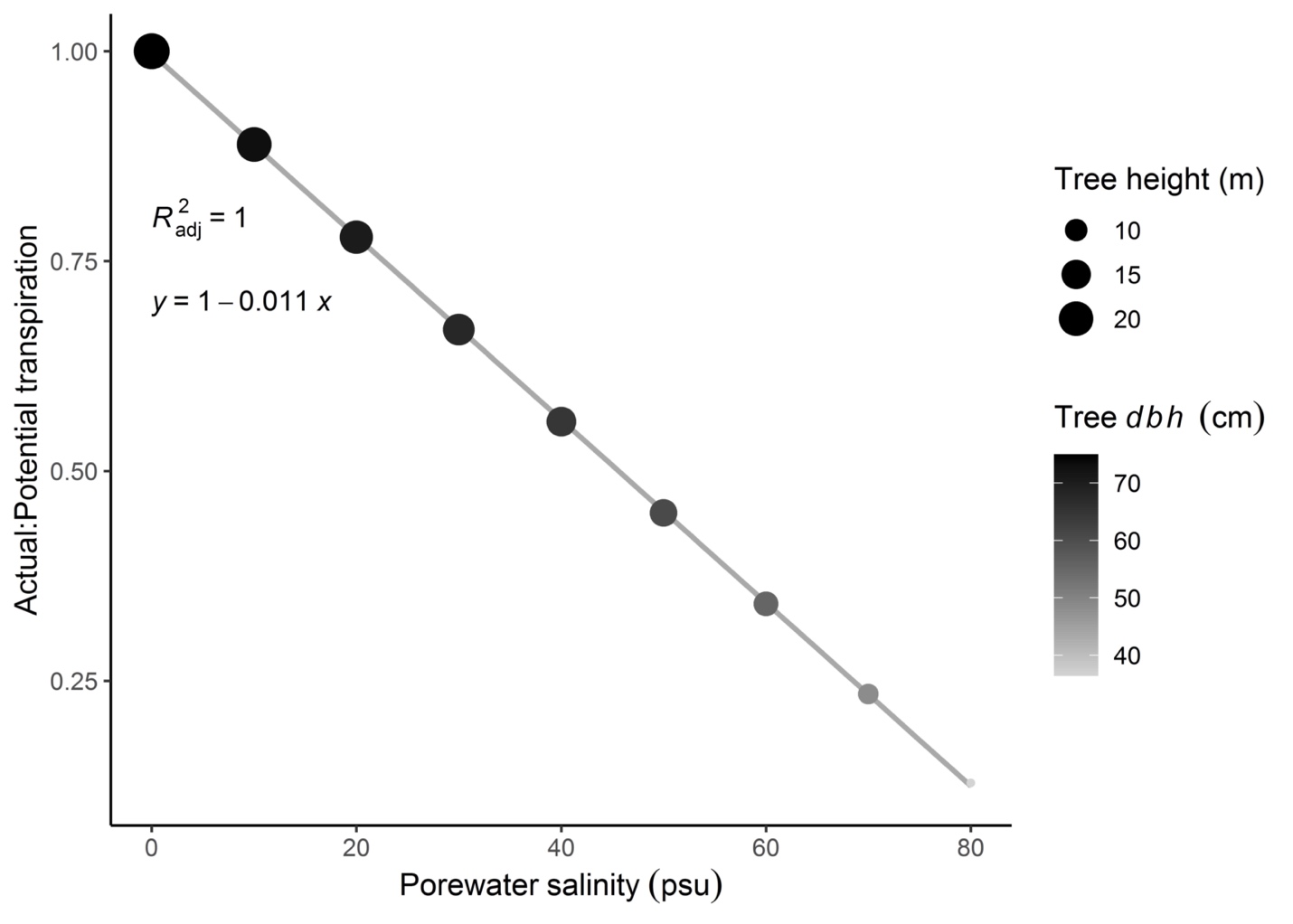


**Figure S3. BETTINA-simulated ratio of actual and potential transpiration of nine mangrove trees (after 200 years for this simulation).** Trees grew under different salinity conditions (0-80 psu), and shades and size of circles represent stem diameter (cm) and tree height (m), respectively.

**Table S1.** Characteristics of 71 sites from 26 published records that report mangrove net primary productivity (NPP), or enough data to calculate NPP using several assumptions, from locations in the Florida-Caribbean Region (N=25) and Asia-Pacific Region (N=46), including calculations of dominant canopy transpiration (*E*_c_) and associated MODIS satellite (MOD16-A3) estimates of potential evapotranspiration (*ET*) for the period of 2000-2013. Global values of 1.03 for were used for wood:litter and 1.20 for root:litter (Bouillon et al.^70^). Canopy transpiration (*E*_c_) was estimated from the predictive regression of, *E*_c_ = (384.5897 × NPP) + 33.5565, where *E*_c_ is in mm H_2_O year^-1^ and NPP equals net primary productivity in kg C m^-2^ year^-1^.

**Table S1.** Continued

**Table S1.** Continued

**Table S2.** Values from 17 categories used to calculate Virtual Water Content for mangroves versus 7 ecoregions to which relative comparisons to mangroves can be made. Currency is reported in U.S. Dollars (USD) for the year 2007. Biome is as reported originally in *Nature*, v. 387, p. 253-260 (1997).

| **Author(s)** | **Date** | **Citation** |
| --- | --- | --- |
| T. J. Andrews & G. J. Muller | 1985 | *Oecologia*, v. 65, p. 449-455 |
| B. F. Clough & R. G. Sims | 1989 | *Oecologia*, v. 79, p. 38-44 |
| J.A.C. Smith, M. Popp, U. Lüttge, et al. | 1989 | *New Phytol.*, v. 111, p. 293-307 |
| S. R. Pezeshki, R.D. DeLaune, & W.H. Patrick, Jr. | 1990 | *Can. J. Forest Res.*, v. 20, p. 869-874 |
| J.M. Cheeseman, B.F. Clough, D.R. Carter, et al. | 1991 | *Photosyn. Res.*, v. 29, p. 11-22 |
| G. Lin & L.d.S.L. Sternberg | 1992 | *Oecologia*, v. 90, p. 399-403 |
| C.E. Martin, & V.S. Loeschen | 1993 | *Photosynthetica*, v. 28, p. 391-400 |
| G. Naidoo, & D.J. von Willert | 1995 | *Hydrobiologia,* v. 295, p. 13-22 |
| K.W. Krauss, R.R. Twilley, T.W. Doyle, & E.S. Gardiner | 1997 | *Tree Physiol.*, v. 26, p. 959-968 |
| S.C. Snedaker, & R.J. Araújo | 1998 | *Mar. Freshw. Res.*, v. 49, p. 325-327 |
| G. Naidoo, H. Rogalla, & D.J. von Willert | 1998 | *Mangroves Salt Marshes*, v. 2, v. 99-107 |
| T. Youssef, & P. Saenger | 1998 | *Mar. Freshw. Res.*, v. 49, p. 329-334 |
| M.A. Sobrado, & M.C. Ball | 1999 | *Aust. J. Plant. Physiol.*, v. 26, p. 245-251 |
| M. Mwangi Theuri, J.I. Kinyamario, & D. Van Speybroeck | 1999 | *African J. Ecol.*, v. 37, p. 180-193 |
| M.A. Sobrado | 2000 | *Trees Struct. Funct.*, v. 14, p. 258-262 |
| L. Chen, N.F.Y. Tam, J. Huang, et al. | 2008 | *Estuar. Coast. Shelf Sci.*, v. 79, p. 644-652 |
| P. Cardona-Olarte, K.W. Krauss, & R.R. Twilley | 2013 | *Int. J. For. Res.*, v. 2013, art. 524625 |

**Table S3.** References for data used to present relationships reported in Figure 1a.

| **Author(s)** | **Date** | **Citation** |
| --- | --- | --- |
| K.W. Krauss, P.J. Young, J.L. Chambers, et al. | 2007 | *Tree Physiol.*, v. 27, p. 775-783 |
| E. Muller, L. Lambs, & F. Fromard | 2009 | *Ann. For. Sci.*, v. 66, 803 |
| L. Lambs, & P. Saenger | 2011 | *Rapid Comm. Mass Spectrom.*, v. 25, p. 2741-2748 |
| K.W. Krauss, K.L. McKee, & M.W. Hester | 2014 | *Ecohydrol.*, v. 7, p. 354-365 |
| K.W. Krauss, J. G. Barr, V. Engel, et al. | 2015 | *Agri. For. Meteorol.*, v. 213, p. 291-303 |
| H. Zhao, S. Yang, X. Guo, et al. | 2018 | *Tree Physiol.*, v. 38, p. 276-286 |
| B. Leng & K.-F. Cao | 2020 | *Glob. Ecol. Conserv*., v. 24, e10233 |
| X. Gu, C. Yang, H. Zhao, et al. | 2021 | *Trees Struct. Funct.*, v. 35, p. 907-917 |

**Table S4.** References for data used to present relationships reported in Figure 1b.

| **Author(s)** | **Date** | **Citation** |
| --- | --- | --- |
| B. Christensen | 1978 | *Aquat. Bot.*, v. 4, p. 43-52 |
| J-.E. Ong et al. | 1979 | *Trends Appl. Biol. S.E. Asia* (Abstract) |
| F.E. Putz, & H.T. Chan | 1986 | *For. Ecol. Manage.*, v. 17, p. 211-230 |
| J.W. Day Jr., W.H. Conner, F. Ley-Lou, et al. | 1987 | *Aquat. Bot.*, v. 27, p. 267-284 |
| D. Imbert, & B. Rollet | 1989 | *Bull. Ecol.*, v. 20, p. 27-39 |
| S.Y. Lee | 1990 | *Mar. Biol.*, v. 106, p. 453-463 |
| J. H. Warner | 1990 | MS Thesis, Univ. Southwestern Louisiana (USA) |
| M.D. Amarasinghe, & S. Balasubramaniam | 1992 | *Hydrobiologia*, v. 247, p. 37-47 |
| S. Sukardjo, & I. Yamada | 1992 | *For. Ecol. Manage.*, v. 49, p. 195-209 |
| J-.E. Ong, G.W. Khoon, & B.F. Clough | 1995 | *J. Biogeography*, v. 22, p. 417-424 |
| J.W. Day Jr., C. Coronado-Molina, F.R. Vera-Herrera, et al. | 1996 | *Aquat. Bot.*, v. 55, p. 39-60 |
| E.F. Cox, & J.A. Allen | 1999 | *Estuaries*, v. 22, p. 276-284 |
| M.S. Ross, P.L. Ruiz, G.J. Telesnicki, et al. | 2001 | *Wetlands Ecol. Manage.*, v. 9, p. 27-37 |
| R.E. Sherman, T.J. Fahey, & P. Martinez | 2003 | *Ecosystems*, v. 6, p. 384-398 |
| S.M.L. Ewe, E.E. Gaiser, D.L. Childers, et al. | 2006 | *Hydrobiologia*, v. 569, p. 459-474 |
| F.L. Lema Vélez, & J. Polanía | 2007 | *Rev. Biol. Trop.*, v. 55, p. 11-21 |
| M. Hossain, S. Othman, J.S. Bujang, et al. | 2008 | *For. Ecol. Manage.*, v. 255, p. 179-182 |
| M.N.I. Khan, R. Suwa, & A. Hagihara | 2009 | *Wetlands Ecol. Manage.*, v. 17, p. 585-599 |
| D.M. Alongi | 2011 | *Estuaries and Coasts*, v. 34, p. 32-44 |
| R. Ray, D. Ganguly, C. Chowdhury, et al. | 2011 | *Atmos. Environ.*, v. 45, p. 5016-5024 |
| S. Poungparn, A. Komiyama, T. Sangteian, et al. | 2012 | *J. Trop. Ecol.*, v. 28., p. 303-306 |
| E. Castañeda-Moya, R.R. Twilley, & V.H. Rivera-Monroy | 2013 | *For. Ecol. Manage.*, v. 307, p. 226-241 |
| K. Fujimoto, R. Tabuchi, Y. Hirata, et al. | 2013 | *Glob. Environ. Res.*, v. 17, p. 207-214 |
| T. Enoki, K. Yasuda, & B. Kusumoto | 2014 | *Tropics*, v. 23, p. 91-98 |
| S. Umnouysin, T. Sangtiean, & S. Poungparn | 2017 | *Ecol. Res.*, v. 32, p. 51-60 |
| A.S. Rovai, C. Coelho-Jr, R. de Almeida, et al. | 2021 | *For. Ecol. Manage.*, v. 479, art. 118553 |

**Table S5.** References for mangrove productivity data identified by location in Figure 3, used for summary calculations reported in Table 1, and summarized in greater detail in Table S1.

**Table S6.** Global mangrove area (from Global Mangrove Watch)^72^ associated with terrestrial ecoregion designations, including how designations were aligned in our determination of scaled water use.

**Table S7.** Raw data extracted from sources and graphed in Figure 1.

**Table S7.** Continued
